# *Thermomonospora* spp. are implicated in the biodegradation of long-chain aliphatic polyester bioplastics during thermophilic composting

**DOI:** 10.3389/fmicb.2025.1671731

**Published:** 2025-10-14

**Authors:** Harry Lerner, Marcel Eck, Christoph Link, Timo Witt, Glauco Battagliarin, Stefan Mecking, David Schleheck

**Affiliations:** ^1^Microbial Ecology and Limnic Microbiology, Department of Biology, Limnological Institute, University of Konstanz, Konstanz, Germany; ^2^Chemical Materials Science, Department of Chemistry, University of Konstanz, Konstanz, Germany; ^3^BASF SE, PMD/GB—B001, Ludwigshafen am Rhein, Germany; ^4^The Konstanz Research School Chemical Biology, University of Konstanz, Konstanz, Germany

**Keywords:** bioplastic, biodegradation, industrial thermophilic composting, ISO 14855-1, bacterial and fungal community compositions, *Thermomonospora curvata*, recombinant polyester hydrolase, depolymerase

## Abstract

Biodegradable plastics are an important component for achieving a circular polymer economy. To be considered biodegradable at the regulatory level, plastics must pass standardized tests, for example under industrial composting conditions at 58 °C (ISO 14855-1). Although such tests are frequently applied, little is known about the microorganisms catalyzing these degradation processes. Recently, bioplastics with properties similar to polyethylene, Long-Chain Aliphatic Polyesters (LCAP), for example polyester 1,18-octadecanediol-*alt*-1,18-octadecanedioic acid (abbreviated PE-18,18), were shown to biodegrade under industrial composting conditions. In this work, we analyzed the microbial communities that had developed in the compost treatments at the end of the biodegradation test for three different LCAPs (PE-18,18, PE-12,12 and PE-2,18) relative to the untreated controls, via amplicon-sequencing of bacterial 16S and fungal ITS2 rDNA. This revealed significant treatment-induced shifts in the bacterial communities (*p* < 0.05), with *Pseudonocardia* and *Thermomonospora* ASVs enriched in all LCAP-treated samples compared to the controls (*p* ≤ 0.0001), while no pronounced shifts were observed for the fungal community. *Thermomonospora* sequences showed high similarity to *T. curvata* DSM43183, which encodes the known polyester hydrolase Tcur1278, and the presence of gene *tcur1278* was confirmed in LCAP-treated samples via PCR. Enzyme assays with heterologously expressed and partially purified Tcur1278 demonstrated its activity on PE-2,18 LCAP, releasing up to 230 μmol of soluble monomers over 48 h at 50 °C. Hence, our study implicated *Thermomonospora* species in LCAP degradation during thermophilic composting, based on taxonomic enrichment, and provided evidence linking the detected phylotypes to Tcur1278, the first bacterial enzyme demonstrated to depolymerize LCAP. It thereby is the first evidence for an ecological relevance of Tcur1278-encoding *Thermomonospora* phylotypes for bioplastic degradation *in situ*.

## 1 Introduction

The ever-increasing accumulation of post-consumer plastic in the environment has received much attention in recent years and inspired industry and academia to intensify efforts to achieve a circular polymer economy. Alongside a sustainable waste hierarchy, including waste prevention and minimization, reuse and repair, followed by recycling and other forms of recovery (e.g., incineration with energy capture), alternatives to the conventional, virtually non-degradable synthetic plastic polymers play a key role in the transition to a sustainable and more circular polymer economy. Biodegradable plastics sourced from renewable materials, such as plant oils, are a promising step toward achieving this goal. However, despite their importance to the circular economy, existing bioplastics have so far failed to emerge beyond a minor market share, calling for further innovation in this field (Abe et al., 2021; Abrha et al., 2022; Boey et al., 2022; Nanda et al., 2022; Rosenboom et al., 2022; González-López et al., 2023; Negrete-Bolagay and Guerrero, 2024; Serrano-Aguirre and Prieto, 2024).

In this context, materials such as long-chain aliphatic polyesters (LCAP) with the potential to replace conventional polyolefins such as high-density polyethylene (HDPE) and polypropylene (HDPP) have evolved. LCAP are comprised of ester-linked long-chain dialcohol (e.g., 1,18-octadecanediol) and dicarboxylic acid (e.g., 1,18-octadecanedioic acid) monomers (thus, polyester-C_18_, C_18_; abbreviated PE-18,18), which can be sourced from plant oils (Häußler et al., 2021). They possess material properties comparable to HDPE, while also exhibiting high-yield and low-energy chemical recyclability (Häußler et al., 2021). Additionally, the biodegradability of LCAP bioplastics was demonstrated under controlled, thermophilic composting conditions according to ISO 14855-1, a standardized test method for the determination of the ultimate aerobic biodegradability of plastics (Eck et al., 2023). Compost samples that derived from this test and that were used for microbial community analysis, are the subject of this communication. Particularly the PE-2,18 variant of LCAP reached a degradation of 96 ± 2% (*n* = 3) within two months at 58 °C, achieving near-complete stoichiometric conversion to CO_2_ despite its polyethylene-like material properties (Eck et al., 2023). This fulfills regulatory requirements for biodegradability (e.g., according to EN 13432), and demonstrates the high potential of LCAP for achieving a circular plastic economy.

Biodegradation of synthetic polymers is influenced by various factors, particularly the chemical structure of the polymer, including the presence or absence of functional groups susceptible to hydrolysis. As such, a meaningful degradation has only been demonstrated for polymers with hydrolysable bonds present in their backbone structure, for example polyethylene terephthalate (PET), polylactic acid (PLA), polycaprolactone (PCL) and polybutylene adipate terephthalate (PBAT), among others (Yoshida et al., 2016; Meyer-Cifuentes et al., 2020; Ru et al., 2020; Teixeira et al., 2021; Buchholz et al., 2022; Gambarini et al., 2022; Lear et al., 2022; Jendrossek, 2024). In addition to the chemical structure of the polymer chain and other factors such as crystallinity of the plastic material, a diverse and adaptable microbial community is key for successful biodegradation, which may differ strongly between habitats (Shah et al., 2008).

The compost matrix contains a vast diversity of naturally occurring microorganisms which are particularly specialized on the utilization of complex plant polymers such as cellulose, lignin and cutin. Notably, enzymes identified to depolymerize synthetic polyesters often share a close homology to those involved in the degradation of natural polyesters, such as the plant hetero-polyester cutin (Chen et al., 2020; Tournier et al., 2020; Sonnendecker et al., 2022; Arnal et al., 2023; Burgin et al., 2024; Ma et al., 2024). Additionally, the high temperatures of up to 70 °C during industrial composting is close to the glass transition temperature (T*g*) of some polymers. These temperatures cause polymer chains in amorphous regions to become more flexible and accessible, enabling increased enzymatic accessibility (Karamanlioglu and Robson, 2013; James-Pearson et al., 2023; Shalem et al., 2024). Therefore, complex soil environments, and thermophilic compost in particular, provide ideal conditions for facilitating the biodegradation of synthetic polymers. This is also evidenced by the frequent isolation of plastic-degrading microorganisms and enzymes from these environments (Buchholz et al., 2022; Gambarini et al., 2022). Particularly thermophilic genera belonging to the Actinomycetota, such as *Thermobifida*, *Streptomyces*, and *Saccharomonospora* have been repeatedly isolated from composting systems and implicated in plastic degradation (Kleeberg et al., 1998; Hu et al., 2010). Other thermophilic actinomycetes, including *Pseudonocardia* and *Thermomonospora*, have been associated with PLA degradation through screening of culture collections or sequencing-based studies, highlighting the potential ecological relevance of this phylum for synthetic polymer degradation (Sangwan and Wu, 2008; Apinya et al., 2015; Butbunchu and Pathom-Aree, 2019). In addition, homology-based genome mining revealed that *Thermomonospora curvata* DSM43183 encodes two putative polyester hydrolases, Tcur1278 and Tcur0390, with high sequence homology to *Thermobifida fusca* polyesterases and demonstrated potential to hydrolyze the plastic materials PET and PCL *in vitro* (Wei et al., 2014). While the ecological relevance of this genotype in polyester degradation has not been demonstrated, other *Thermomonospora* strains isolated from soil, compost, manure, and plant material were reported to degrade natural polymers such as cellulose and rubber latex, indicating their ecological relevance in natural polymer degradation (Henssen, 1957; Ibrahim et al., 2006; Chertkov et al., 2011).

Nevertheless, synthetic-polymer biodegradation studies employing compost are commonly operated as a microbial ‘black-box’. While the standardized test employs standardized incubation conditions (e.g., 58 °C, aeration, moisture control, as for test ISO 14855-1; see below), the individual studies are being conducted with different compost matrices that may contain vastly different microbial communities. Therefore, advancing our ecological understanding of plastic biodegradation in these systems is important not only for validating biodegradation claims, but also for informing material design and developing improved strategies for sustainable waste processing.

To date, few studies have attempted to directly identify the microorganisms involved in the degradation process during thermophilic composting, with varying results. While one study identified clear microbial community shifts in response to biodegradable plastic treatments, other studies also reported significant shifts in HDPE treatments in absence of observable degradation (Esan et al., 2019; Sun et al., 2021; Ruggero et al., 2023). These results demonstrate the challenge of assessing whether plastic-dependent community shifts are in fact associated with polymer degradation, or whether taxa simply make use of an additional colonizable surface (the ‘plastisphere’), especially with respect to plastics widely regarded as non-degradable (Wallbank et al., 2022). Therefore, inferring an organism’s involvement in degradation processes from an increased occurrence in the plastisphere or throughout the overall community might result in erroneous assignment of ecological relevance. As such, studies often lack a link between substrate-specific community shifts in response to plastic exposure and the catabolic potential of candidate taxa to facilitate the observed degradation. Consequently, still relatively little is known about organisms actually catalyzing the *in-situ* degradation of synthetic polymers, particularly in the context of controlled composting.

In the present study, we report the first microbial community analysis of an LCAP-degrading environment, i.e., thermophilic compost. We aimed not only to identify taxa likely involved in LCAP degradation based on treatment-specific abundance increases, but also to provide functional evidence of their capacity to facilitate the observed degradation process. To this end, we compared the microbial communities across end-point samples of compost material used in the previously reported study of LCAP biodegradation. The composting experiment, as described by Eck et al. (2023), included the LCAP bioplastic materials PE-18,18 (polyester of octadecane-1,18-diol and 1,18-octadecanedioic acid) and PE-12,12 (polyester of dodecane-1,12-diol and 1,12-dodecanedioic acid) and PE-2,18 (polyester of ethane-1,2-diol [ethylene glycol] and 1,18-octadecanedioic acid), and an untreated compost sample as control, each in triplicate incubations.

The microbial community analysis revealed a significant increase of *Pseudonocardia* and *Thermomonospora* ASVs in response to LCAP treatment. We provide additional evidence of the functional capacity of *Thermomonospora* phylotypes to facilitate LCAP depolymerization, by detection of gene homologs of the *Thermomonospora curvata* polyester hydrolase Tcur1278 (Wei et al., 2014) in the LCAP-degrading reactors by PCR. Additionally, *in-vitro* testing of this enzyme demonstrated its ability to depolymerize LCAP. Hence, the taxonomic, genetic, and functional evidence combined appoints *Thermomonospora* as a likely *in-situ* degrader of LCAP in the investigated environment.

## 2 Materials and methods

### 2.1 Samples of bioplastic-degrading compost material

The compost samples used in this study originate from the reactors used in a preceding biodegradation study according to ISO 14855-1 (Eck et al., 2023). Representative samples were collected from each of the triplicate composting reactors by thoroughly mixing the reactor contents and taking one 5–10 g sample per reactor (n = 3 per treatment). Samples were collected and flash-frozen in liquid N₂ immediately after the 66-day incubation and stored at −80 °C until DNA extraction.

The composting experiment, as described by Eck et al. (2023), included the long-chain aliphatic polyester (LCAP) bioplastic materials PE-2,18 (polyester of ethane-1,2-diol [ethylene glycol] and 1,18-octadecanedioic acid), PE-18,18 (polyester of octadecane-1,18-diol and 1,18-octadecanedioic acid) and PE-12,12 (polyester of dodecane-1,12-diol and 1,12-dodecanedioic acid), which were synthesized in-house and cryo-milled to a particle size of 100–300 μm. For each treatment, 8 g of cryo-milled polymer powder was mixed into 120-g portions of compost (46.5% water content) in 1 L glass bottles. The bottles were sealed with caps equipped with an air inlet and outlet, allowing moisture to be maintained during incubation by flushing with air at 100% relative humidity. Incubations were conducted in a temperature-controlled incubator at 58 ± 2 °C. Cellulose powder was used as a reference material, and compost without polymer served as a control. Each treatment was set up in triplicate and incubated for 66 days. For further details pertaining to the composting experiment and the plastic materials used, see Eck et al. (2023).

### 2.2 Bacterial strains

*Thermomonospora curvata* DSM43183 was purchased from the German Collection of Microorganisms and Cell Cultures (DSMZ, Leipzig, Germany) as an actively growing culture on starch mineral salts agar (DSMZ medium 252). The organism was sub-cultivated and maintained in the lab using DSMZ medium 550 or nutrient agar. *E. coli* expression strain Rosetta-Gami 2 (DE3) was maintained in cryo-stocks and propagated using LB medium.

### 2.3 DNA extraction and PCR

Total DNA in compost samples was extracted using a cetyltrimethylammonium bromide (CTAB)-based method adapted from Larsen et al. (2007). To avoid PCR inhibition by soil constituents such as humic acids, the DNA was cleaned further using polyvinylpolypyrrolidone spin-columns before down-stream applications (Berthelet et al., 1996). DNA of *T. curvata* DSM43183 was extracted using the Monarch Genomic DNA Purification Kit (New England Bio Labs Inc., Ipswich, MA, USA), following the manufacturer protocol for Gram-positive bacteria and archaea.

A fragment of the 16S rRNA gene was amplified by PCR using primers Bakt341F (CCTACGGGNGGCWGCAG) and Bakt805R (GACTACHVGGGTATCTAATCC), targeting the V3 and V4 region of the gene (Klindworth et al., 2013). The internal spacer region 2 (ITS2) was amplified using primers fITS7 (GTGARTCATCGAATCTTTG) and ITS4 (TCCTCCGCTTATTGATATGC) (Purahong et al., 2021). The primers were equipped with the appropriate adapters for Illumina sequencing platforms. PCR reactions and purification of the amplicons was performed as recommended in the “16S Metagenomic Sequencing Library Preparation” protocol (Illumina, SanDiego, CA, USA). For amplification of the Tcur1278 encoding gene, the primer pair Tcur1278-F (GATCCCACCGAATCCCTTCT) and Tcur1278-R (CGTTGGTCAGGCTGTTGTAG) was designed using primer3 and its specificity validated *in silico* using primer Blast (Untergasser et al., 2012; Ye et al., 2012). The PCR reaction for the amplification of *tcur1278* was carried out using Phusion High Fidelity Polymerase (New England Biolabs, Ipswich, MA, USA), with initial denaturation at 95 °C for 3 min, followed by 30 cycles of 30 s denaturation at 95 °C, 30 s annealing at 55 °C, and 40 s elongation at 72 °C. The reaction was concluded with a final elongation step of 5 min at 72 °C.

### 2.4 PCR amplicon sequencing and phylogenetic analysis

Purified amplicons were sent to Eurofins genomics (Konstanz, Germany) for library preparation and paired-end sequencing on an Illumina MiSeq platform. The sequencing reads were analyzed as previously described (Lerner et al., 2020), except that the latest versions of the respective software packages were used. Briefly, the raw reads were quality filtered, trimmed and chimeric sequences were removed utilizing the package dada2 (v.1.26.0) in R (v.4.3.2) (Callahan et al., 2016; R: A language and environment for statistical computing, 2024). Taxonomy was assigned to ASVs using the Silva database version 138 for bacterial 16S rRNA gene amplicons and the UNITE ITS database version 9 for fungal ITS amplicons (Quast et al., 2013). Processing of 16S rRNA gene and ITS amplicon sequences was identical, the only difference being that ITS reads were not filtered based on sequence length, due to the biological length variation of the ITS region.

Microbial community analysis was performed in R using packages pyhloseq (v.1.42.0) (McMurdie and Holmes, 2013), Biostrings (v.2.66.0), (Pagès et al., 2019) ape (v.5.7.1), and vegan (v.2.6–6.1) (Oksanen et al., 2024) Log_2_-fold change analysis (lfc) was performed using R package ANCOMBC2 (v.2.0.2) with “struc_zero” set to FALSE (Lin and Peddada, 2020; Lin et al., 2022). Prior to lfc analysis, ASVs with less than 10 counts across 10% of samples were excluded to avoid over-estimation of rare taxa in the analysis. R packages ggplot (v.3.4.2) and ggsci (v.3.0.0) were utilized in data visualization (Wickham, 2016).

The maximum likelihood phylogram of *T. curvata* associated 16S rRNA gene sequences in relation to additional sequences downloaded from the NCBI nucleotide database, was obtained following alignment using MUSCLE (v3.8.1551) and subsequent 1,000x bootstrap analysis using RAxML (v.8.2.12), under implementation of the GTRGAMMA nucleotide substitution model^,^(Edgar, 2004; Stamatakis, 2014). The Phylogenetic tree was visualized using TreeViewer (v. 2.2.0) (Bianchini and Sánchez-Baracaldo, 2024).

### 2.5 Heterologous expression and purification of Tcur1278 protein

A pET-26b(+) vector containing *tcur1278* followed by a C-terminal His-tag was purchased from BioCat (Heidelberg, Germany). The gene sequence optimized matching the preferred codon usage of *E. coli* was inserted without its PelB secretion signal to enable the intracellular accumulation of the heterologous enzyme. The vector was transformed into *E. coli* expression strain Rosetta-Gami 2(DE3) using heat-shock transformation. The expression strain was incubated in a 250 mL baffled flask containing 50 mL of LB medium with 50 μg ml^−1^ Kanamycin (Kn) and grown at 37 °C in a horizontal shaker at 200 rpm overnight. The 50 mL culture was transferred into 1 L of LB_Kn50_ medium in a 5 L baffled flask and incubated for 2–4 h at 37 °C until an OD_600_ of 0.6–0.8 was reached. The culture was then briefly cooled on ice and expression was induced by addition of IPTG to a final concentration of 0.4 mM. The incubation was continued and Tcur1278 expressed at 18 °C and 80 rpm of horizontal shaking for up to 24 h. Cells were harvested by centrifugation at 5,000 x *g* for 15 min and the pellet washed twice with 50 mM sodium phosphate buffer containing 300 mM NaCl (pH 7.4). The cell pellet was frozen in liquid N_2_ and stored at −80 °C until preparation of cell extracts.

The frozen pellet was thawed, and cells were resuspended in 30 mL lysis buffer containing 50 mM sodium phosphate buffer (pH 8.5), 300 mM NaCl, 0.1% Triton-X100, 10 mM imidazole and EDTA-free protease inhibitor (Roche, Basel, Switzerland). Cell lysis was achieved through sonification on ice for 8 min using a SONOPLUS HD 2070.2 with VS70T (BANDELIN, Berlin, Germany) operated at 25% amplitude, 1 s active, 3 s pause. The lysate was centrifuged at 16,000 x *g* for 30 min and the soluble protein fraction was partially purified in a 1-mL HisTrap column (Cytiva, Uppsala, Sweden) according to the manufacturer’s specifications, using concentrations of 30 mM and 500 mM imidazole for the wash and elution step, respectively. Imidazole was removed from the eluate using Amicon centrifugal filters (10 kDa, Merck, Darmstadt, Germany). Expression and purity of the target protein were evaluated via SDS-PAGE. Protein concentration in the partially purified fraction was determined via Bradford assay (Bradford, 1976).

### 2.6 Depolymerase enzyme assays

Depolymerization of PE-2,18 and PCL by heterologously produced and partially purified Tcur1278 was determined in a discontinuous enzyme assay by monitoring the formation of plastic monomers. PE-2,18 was chosen to determine Tcur1278 activity on LCAP due to the water- soluble nature of its C2-monomer, ethylene glycol, allowing for a simple sampling approach. In contrast, the C18 and C12 monomers from other LCAP variants require additional solvent extraction and LC–MS based detection methods. The reactions were conducted in 1.5 mL reaction tubes with 1.4 mL 50 mM sodium phosphate buffer (pH 8.5) containing 0.2 mg mL^−1^ of Tcur1278 and 20 mg of cryo-milled PE-2,18 powder or PCL powder as reference substrate. Triton-X 100 was added to a concentration of 0.01% to facilitate the dispersion of the hydrophobic polymer powders. Reactions were set up in triplicate and controls containing the respective polymer powder but no enzyme were used as negative controls. The reaction mixtures were placed in a heating block at 50 °C with 300 rpm of horizontal shaking. Sub-samples of 200 μL were taken at the start of incubation and after 0.5, 1, 3.5, 4.5 and 24 h, and for the assays with PE-2,18, after 48 h. The reaction in the sub-samples was stopped by the addition of 20 μL 1 M H_2_SO_4_ and the remaining polymer was removed by centrifugation. The supernatant was analyzed via HPLC (LC-20 system, Shimadzu, Kyoto, Japan) for the monomers ethylene glycol (EG) and *γ*-caprolactone (CL), of PE-2,18 and PCL, respectively, as detected with a refractive index detector (RID-20A, Shimadzu, Kyoto, Japan) after separation on a Rezex RHM-monosaccharide ion exchange column (Phenomenex, Torrance, CA, USA) operated at 40 °C with 30 mM H_2_SO_4_ as mobile phase at a flowrate of 0.6 mL min^−1^. Monomers were quantified in reference to concentration series of authentic standards using the Shimadzu Lab Solutions software version 5.81.

### 2.7 Cultivation attempts with *T. curvata* DSM43183 and LCAP

Cultivation attempts with *T. curvata* DSM43183 and PE-12,12 powder were conducted in mineral salts medium buffered with 50 mM potassium phosphate (pH 7.2), containing no added carbon sources other than 0.05% yeast extract, which served to supplement potentially essential growth factors rather than act as a carbon source (Thurnheer et al., 1986). Additional cultivations were conducted in carbon-reduced (by omission of sucrose) DSMZ medium 550 with the addition of PE-12,12 powder in place of other carbon sources. The cultures were set up in 50-mL Erlenmeyer flasks equipped with side arms and lateral reaction tubes (Labor Ochs, Bovenden, Germany) containing 20 mL of the respective medium and 20 mg cellulose or PE-12,12 powder, or no additional carbon source. The cultures were inoculated (1% v/v) with *T. curvata* pre-grown in nutrient broth and after washing with carbon-free mineral salts medium, while the reaction tubes were filled with 4 mL of 0.5 M NaOH to trap all CO_2_ produced. The flasks and reaction tubes were closed with butyl rubber stoppers and incubated at 50 °C, while the culture was agitated by magnetic stirring bars. The NaOH solution was regularly sampled and replaced over the incubation period of 21 days. The carbonate content in the NaOH samples was analyzed using a TOC-L device (Shimadzu, Kyoto, Japan), as previously described (Avasthi et al., 2024).

In addition, a bioaugmentation assay was set up in the same side-arm flasks, containing 10 g forest soil (humus) collected at the Botanical Gardens of Konstanz University (Konstanz, Germany), which was autoclaved twice and supplemented with 30 mg of cellulose or PE-12,12; a control without added polymer was included. *T. curvata* cells were pre-grown in nutrient broth, washed with mineral salts medium and added to the flasks. Additional sterile water was added to reach a moisture content equivalent to 70% of the soil’s water holding capacity. 0.5 M NaOH was added to the reaction tubes as described above and the flasks incubated at 50 °C without agitation for 21 days. NaOH was sampled and analyzed for carbonate content as described above.

## 3 Results

### 3.1 Phylogenetic analysis of compost microbiota after incubation with LCAP powders

This study builds on the work of Eck et al. (2023), who demonstrated mineralization of several LCAP bioplastics under industrial composting conditions (58 °C) using a down-scaled ISO-14855-1 assay. Among the tested polymers, PE-2,18 mineralized rapidly, whereas PE-18,18, and also PE-12,12 (not reported in their study), showed slower degradation. Cellulose was used as a positive control, while reactors without polymer addition served as negative controls. At the end of those incubations, compost samples (5–10 g per reactor) were collected and used in the present study for phylogenetic analysis to investigate treatment-dependent shifts in bacterial and fungal community composition, by 16S rRNA gene-fragment and ITS amplicon sequencing, respectively.

**Figure 1:**
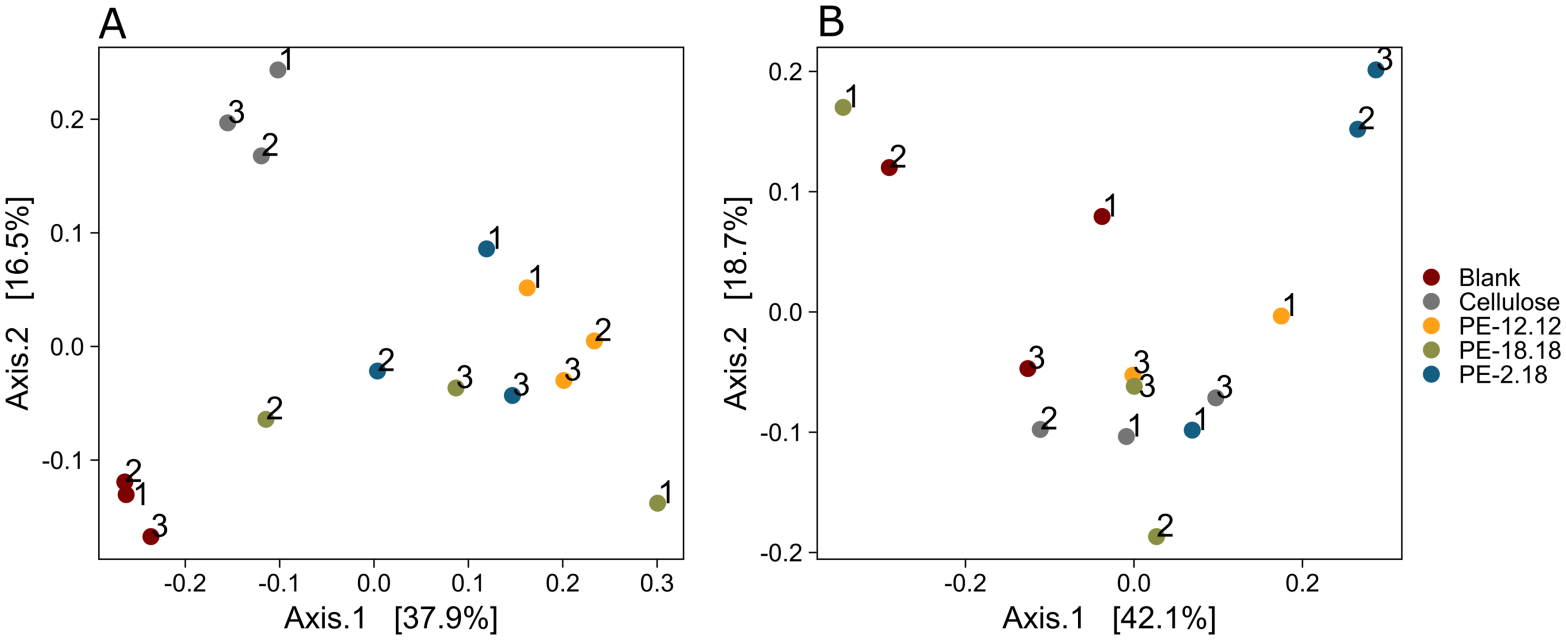
**(A,B)** Principal coordinates analysis (PCoA) of the sequenced bacterial 16S **(A)** and fungal ITS2 **(B)** amplicons, based on Bray–Curtis dissimilarity of the respective ASV datasets. Polymer treatments of the compost materials are indicated by different colors. Numbers next to the datapoints indicate the replicate number. Percentage at the axes indicate the proportion of dissimilarity explained by the respective axis.

Rarefaction curves of the 16S rDNA datasets showed full asymptotes, indicating robust taxonomic sampling (Supplementary Figure S1). The principal coordinates analysis (PCoA) exhibited a clear clustering according to the sample treatment, with the triplicate samples of the blank and cellulose treatments occupying opposite ends of axis 2 in the negative space of axis 1 of the PCoA, while all LCAP-treated samples (PE-2,18, PE-18,18 and PE-12,12) clustered distinctly in the positive space of axis 1, with the exception of replicate 2 of the PE-18,18 treatment (Figure 1A). The significance of the observed clustering was confirmed via PERMANOVA (*p* = 0.001). In contrast, the analysis of the fungal ITS2 amplicons revealed no clear clustering in the PCoA (Figure 1B) and no significant community shifts induced by the different polymer treatments (PERMANOVA: *p* > 0.05). Therefore, the results suggested a more pronounced treatment-specific effect on the bacterial community than on the fungal community, indicating a substrate-induced community shift, potentially associated with the increased abundance of degraders.

**Figure 2:**
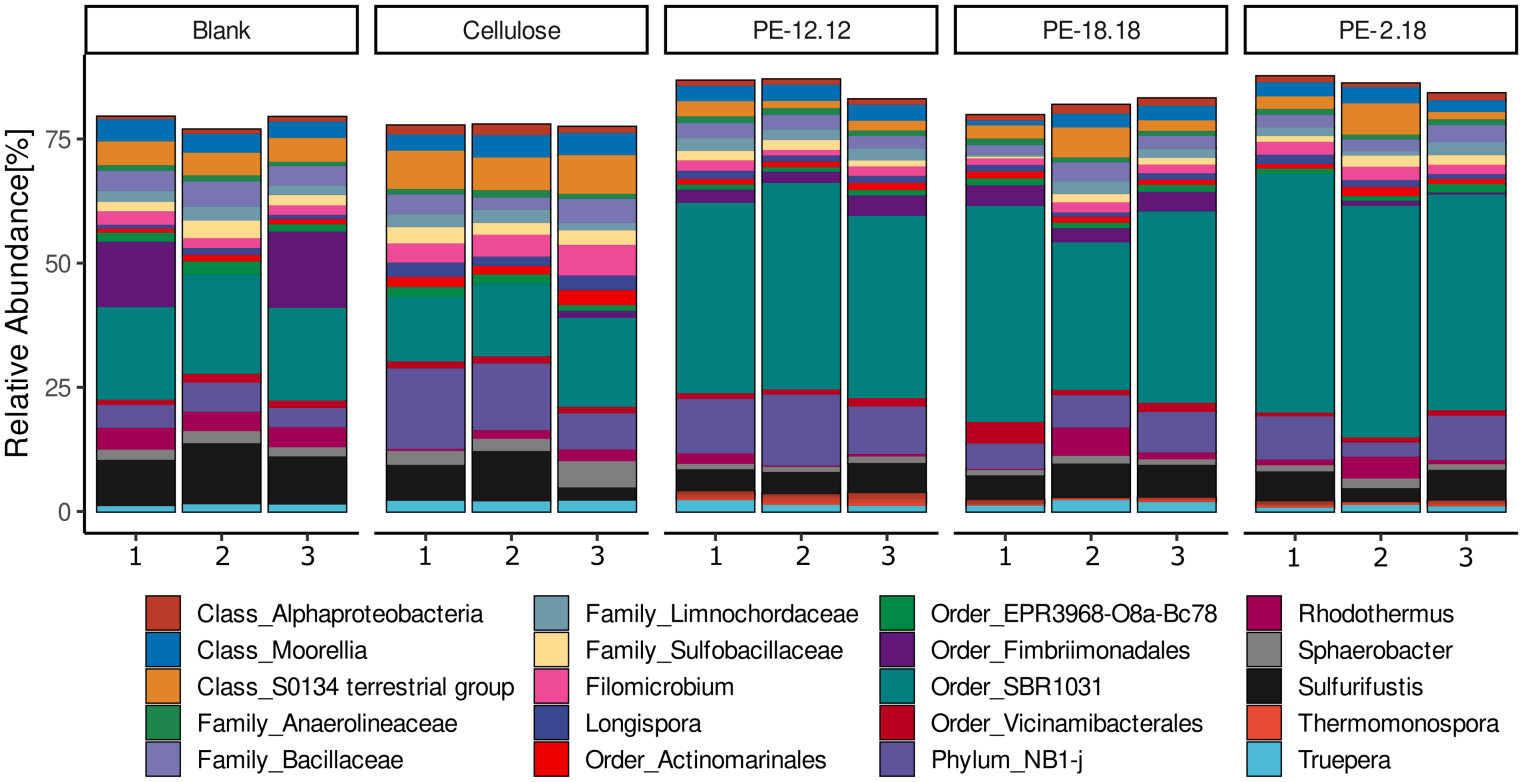
Relative abundances of sequenced 16S rDNA amplicons classified at the genus level. ASVs assigned to the same genus were combined prior to visualization. Only taxa with a relative abundance of at least 0.5% are displayed. Taxa that could not be classified at the genus level with sufficient confidence were classified to the highest possible rank, as indicated accordingly. Polymer treatments are indicated above the bar graphs and the numbers on the x-axis correspond to the three replicates of the respective treatment.

The assessment of all bacterial ASVs at the genus level (Figure 2) did not reveal any evident changes in the overall community structure as a result of the compost treatments. A similar community composition was observed for the most abundant ASVs, most of which were assigned to uncultured taxa, such as the order SBR1031 (class Anaerolineae) and phylum NB1-j, which together contributed between approximately 30–60% across all samples. Other major taxa included the order Fimbriimonadales (formerly phylum OP10) or an unclassified genus of the S0134 terrestrial group of the Gemmatimonadota phylum (Figure 2). However, a bacterial genus that was found to be uniquely represented in all of the LCAP-treated compost reactors while absent in the cellulose and blank controls, was *Thermomonospora* (Actinomycetia), with relative abundances in the range from 0.4% up to 2.6% for one of the replicates of the PE-12,12 treatment (Figure 2).

**Figure 3:**
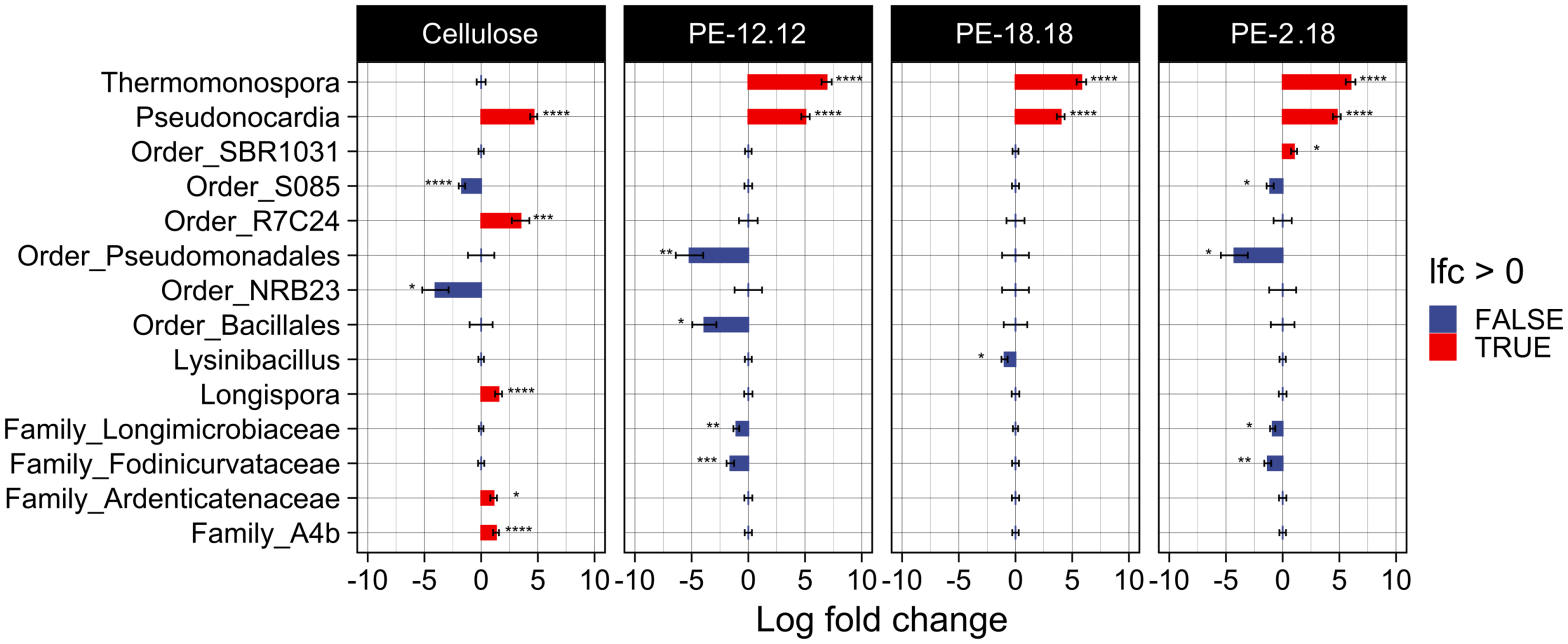
Visualization of the log2-fold change (lfc) analysis of the bacterial communities as determined by 16S rDNA amplicon sequencing and compared to the blank. Only ASVs with more than 10 counts across 10% of samples were considered in order to reduce noise. The analysis was conducted under the inclusion of structural zeroes, i.e., complete absence of an ASV was considered to be a true absence instead of a sampling bias. A statistically significant increase of an ASV is indicated with a red bar, a significant decrease with a blue bar. The taxonomic affiliation of the ASV is indicated. Significance levels are indicated next to respective bar as follows: *p ≤ 0.05, **p ≤ 0.01, ***p ≤ 0.001, and ****p ≤ 0.0001.

Changes in ASV abundances relative to the different compost treatments were further analyzed based on log_2_-fold changes (lfc) (Figure 3). To avoid an over-estimation of rare taxa, ASVs with less than 10 counts across 10% of samples were removed prior to analysis. The lfc analysis confirmed the above-mentioned observation for the *Thermomonospora*-associated ASVs, i.e., their unique presence in samples of PE-2,18-, PE-18,18- and PE-12,12-treated compost (cf. Figures 2, 3). In addition, the lcf analysis revealed more taxa in the LCAP and cellulose treatments that were significantly differing in abundance compared to the blank. ASVs of the genus *Pseudonocardia* (Actinomycetia), for instance, also showed increased abundance in the LCAP-treated compost, but in contrast to *Thermomonospora*, this genus was also significantly increased in the cellulose treatment. Despite the assigned significance, however, the relative abundance of *Pseudonocardia* ASVs in all LCAP-treated samples was lower compared to the *Thermomonospora* ASVs (≤ 0.3%). This difference was also reflected in the significance levels assigned to the individual ASVs in the lfc analysis (Supplementary Figure S2). Furthermore, non-cultivated order R7C24 (Gammaproteobacteria), genus *Longispora* (Actinomycetia), family Ardenticatenaceae (thermophilic Choroflexota) and family A4b (Choroflexota) bacteria showed increased abundance in the cellulose treatment, but not in the LCAP treatments. ASVs assigned to the order Pseudomonadales (Gammaproteobacteria) decreased in relative abundance for the PE-12,12 and PE-2,18, but not for the PE-18,18 treatment. Likewise, ASVs of the order Bacillales and order NRB23 (Clostridia) decreased solely in the PE-12,12, but not in the PE-2,18 and PE-18,18-treatments, and order NRB23 for the cellulose treatment (Figure 3).

**Figure 4:**
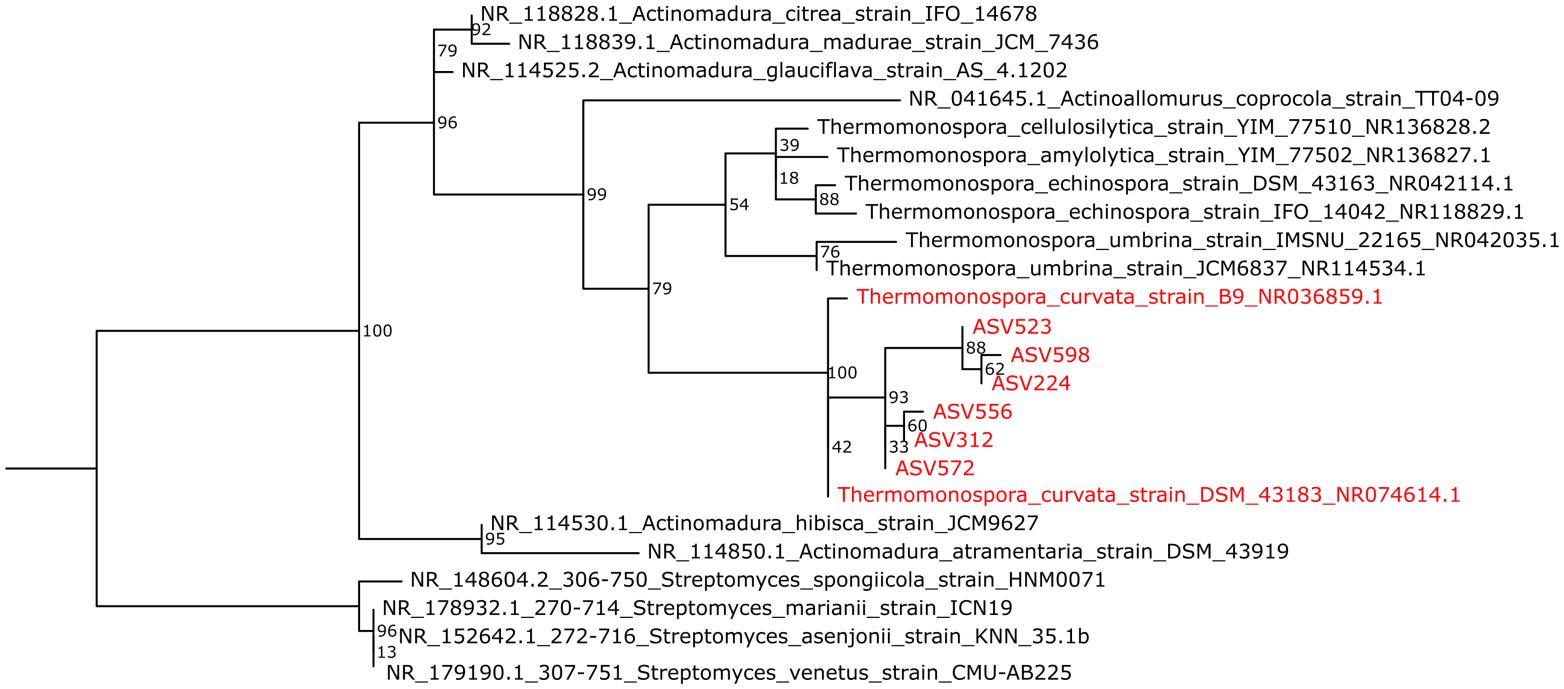
Maximum-likelihood phylogenetic tree showing the 16S rRNA gene ASVs affiliated to *Thermomonospora* species as obtained from LCAP-treated composts (red) and sequences of the *T. curvata* strains B9 and DSM43183 (also red), in relation to sequences of other *Thermomospora* species and of closely related *Actinomadura* species (black). More distantly related sequences of *Streptomyces* species were included for improved clustering (also black). Bootstrap values are indicated at the branch root.

To provide additional taxonomic information, the six 442-bp long ASVs assigned to the *Thermomonospora* genus were aligned to the NCBI RefSeq RNA database using the NCBI BLAST online tool. The best matches showed highest sequence similarity (98.83 ± 0.42%) to the 16S rRNA gene of the type strain *Thermomonospora curvata* DSM43183 (Chertkov et al., 2011). The taxonomic affiliation of the most abundant *Thermomonospora* ASVs to *T. curvata* was further supported by the observed clustering of the sequences in a maximum-likelihood phylogenetic tree (Figure 4), including all available 16S rRNA gene sequences of *Thermomonospora* isolates in the NCBI representative genomes database, as well as sequences of closely related organisms such as those belonging to the *Actinomadura* genus; several *Streptomyces* sequences served as the outgroup for improved clustering (Figure 4).

Hence, the sequencing results indicated an increase in relative abundance of *T. curvata-*associated phylotypes in all compost samples supplied with LCAP materials as additional substrates (Henssen, 1957; Ibrahim et al., 2006; Chertkov et al., 2011).

The treatment-specific increase in relative abundance of *Thermomospora* ASVs and their close phylogenetic relationship to *T. curvata* DSM43183, which encodes polyester hydrolases, strongly suggest a role in LCAP degradation. However, these results do not assign the same functional capacity to the identified phylotypes. Furthermore, while *T. curvata* hydrolases were shown to hydrolyze PET and PCL, their capacity to hydrolyze LCAP is not implied. Therefore, further analyses were conducted to strengthen this conclusion.

### 3.2 Cultivation attempts with *T. curvata* DSM43183 and LCAP and detection of the Tcur1278 polyester hydrolase gene in the studied compost material

The amplicon-sequencing based community analysis strongly suggested the involvement of phylotypes closely related to *T. curvata* in the degradation of LCAP. In absence of *Thermomonospora* isolates from the compost itself, *T. curvata* type strain DSM43183 was used to assess the LCAP-degradation potential of the this strain, and potentially of the closely related *Thermomonospora* spp. present in the investigated compost samples.

The strain was cultivated and maintained using nutrient broth or DSM medium 550. However, repeated attempts at cultivation using defined mineral salts medium supplemented with LCAP or cellulose powder as the sole carbon source were unsuccessful (data not shown). The reason for the unsuccessful cultivation using polymeric substrates is unclear but is most likely linked to the cultivation conditions. In particular, the lack of cellulose degradation suggests a systemic issue with the cultivation approach or problems with specific media components, as cellulose is a well-documented substrate for this strain. In further attempts, soil microcosms were prepared using sterilized (autoclaved) soil, which were spiked with LCAP or cellulose and augmented with pre-grown cultures of *T. curvata* DSM43183. These attempts likewise did not result in observable polymer degradation. Again, it is not clear why no degradation occurred, especially for cellulose. It is possible that the provided soil matrix was not suitable in its nutrient composition, or that microbial interactions are essential for polymer degradation by this strain under the tested conditions. Overall, the cultivation attempts did not provide a clear indication of the degradative potential of this strain, given that even degradation of the known substrate cellulose could not be observed.

We then aimed at investigating the presence of *tcur1278*, the gene encoding the polyester hydrolase of *T. curvata* DSM43183, in the total DNA obtained from the compost samples. Tcur1278 was chosen for testing over Tcur0390 due to its reportedly higher heat tolerance, which, considering the high temperatures during industrial composting, made it the more interesting candidate (Wei et al., 2014). PCR primers targeting *tcur1278* were designed and their specificity confirmed *in silico* and in PCR reactions with strain DSM43183 chromosomal DNA as a template. The qualitative PCR test for the presence of *tcur1278* was positive for the PE-2,18 and PE-12,12 treatments, while no PCR product was detected in the cellulose-treated, non-treated controls, or the PE-18,18 treatments, under the PCR conditions used (Supplementary Figure S3). This result was interpreted to suggest a higher abundance of *tcur1278* homologues in the PE-2,18 and PE-12,12 treatments; however, alternative explanations such as lower gene abundance below the detection limit, sequence divergence affecting primer binding, or differences in DNA template quality cannot be excluded.

### 3.3 Polyester hydrolase Tcur1278 is also able to depolymerize PE-2,18 *in vitro*

**Figure 5:**
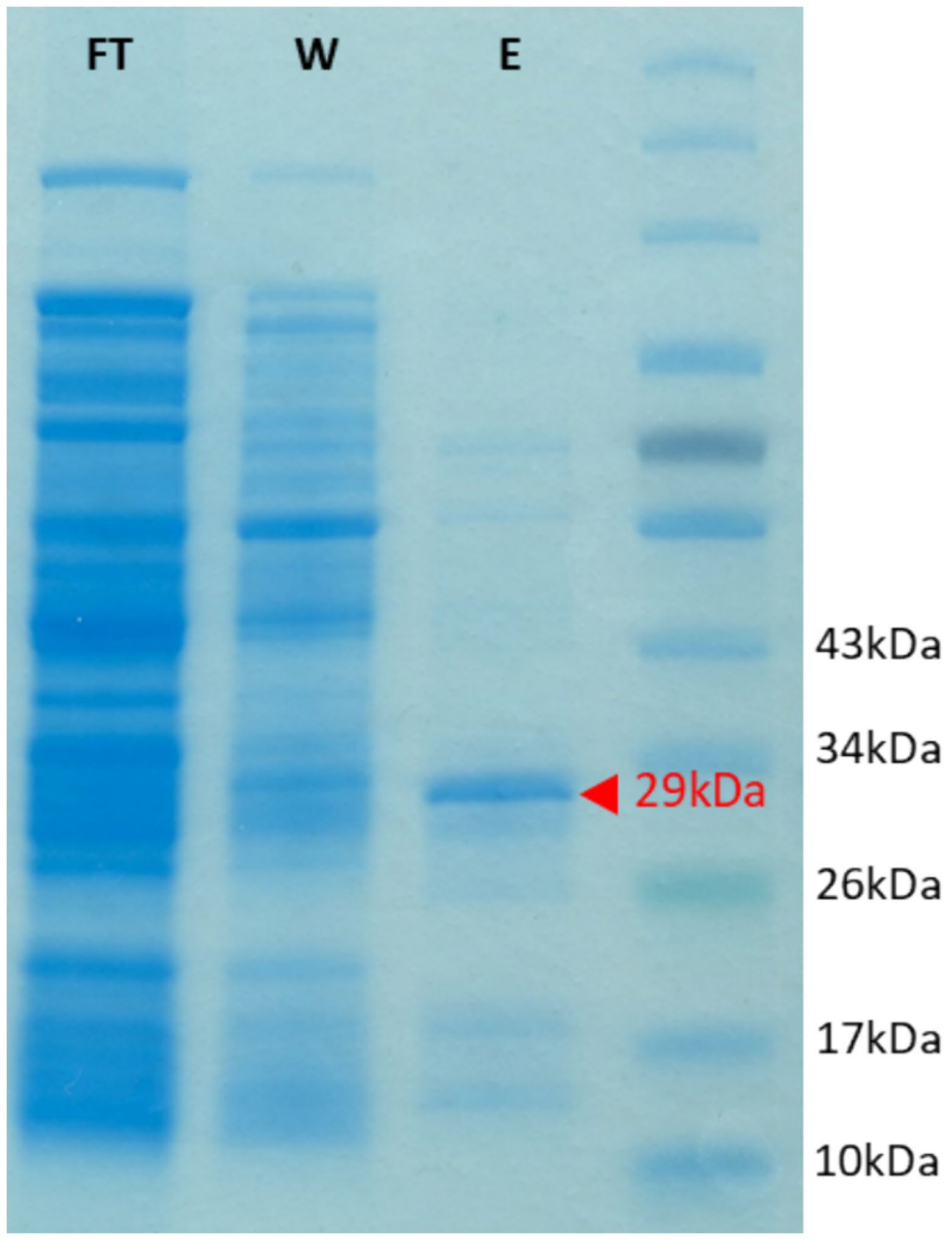
SDS gel showing the heterologous expression of the Tcur1278 protein in *E.coli* Rosetta Gami(DE3). The gel was loaded with protein samples obtained after the loading of the His-Trap column with cell-free lysate (flow-through, FT), after the washing of the column with 30 mM imidazole (wash, W) and of the eluate as obtained after applying 500 mM imidazole to the column. A protein marker was loaded in the final lane for molelular-weight reference. The band corresponding to the Tcur1278 protein is indicated by a red arrow.

We were interested in examining the potential of the characterized PET/PCL-polyester hydrolase Tcur1278 to also hydrolyze LCAP materials. Therefore, Tcur1278 was expressed in Rosetta-Gami 2(DE3) and the expression and partial purification of the protein was confirmed via SDS-PAGE. The expression and partial purification yielded a band with a molecular weight of approx. 29 kDa corresponding to Tcur1278, along with minor contaminating bands (Figure 5).

**Figure 6:**
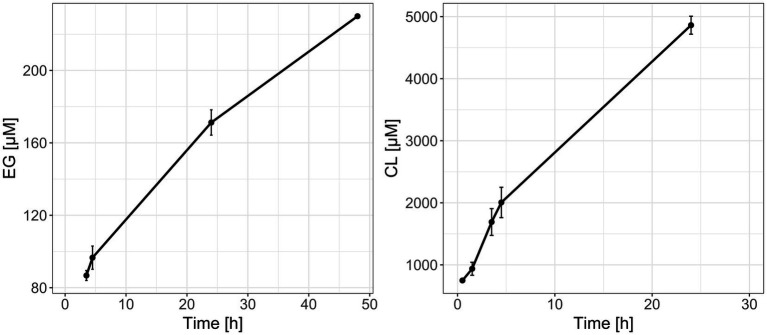
Formation of ethylene glycol (EG, **left panel**) and caprolactone (CL, **right panel**) monomers during reactions of recombinantly produced and purified Tcur1278 polyester hydrolase with PE-2,18 and PCL powder, respectively, as determined by HPLC-RI. Error bars indicate the standard deviation between the triplicate reactions.

Discontinuous enzyme assays with partially purified Tcur1278 and PE-2,18 or PCL showed monomer formation due to enzymatic depolymerization, while no peaks for monomers were detectable in the controls without enzyme. The depolymerization of PCL proceeded more rapidly, yielding an approx. 25-fold higher monomer concentration compared to the PE-2,18 incubations after the same incubation time (Figure 6). The initial measurement of PE-2,18 incubations was below the detection limit and is not depicted. The lower degradation rate of PE-2,18 prompted increased intervals for the measurements to allow detection of maximum monomer formation. Due to the insoluble and slowly degrading nature of the LCAP substrate, classical enzyme kinetic parameters such as Km and Vmax could not be determined.

## 4 Discussion

A sequencing-based analysis of compost samples derived from the ISO14855-compliant LCAP degradation study (Eck et al., 2023) revealed treatment-specific shifts in the bacterial community composition, with significantly increased relative abundance of the actinomycetes *Pseudonocardia* and *Thermomonospora* in all LCAP-treated samples. The analysis of the fungal community, in contrast, revealed no significant treatment-specific community shifts, indicating a more pronounced effect of polymer treatment on the bacterial community. While these results do not explicitly exclude fungi from the observed degradation process, the data are less likely to reveal the potentially involved organisms.

The observed abundances of *Pseudonocardia* and *Thermomonospora* in the LCAP-treated compost were relatively low despite their significant increase compared to the controls (0.3–2.6%), while the bacterial communities overall exhibited remarkably similar core communities, regardless of the applied treatment (Figure 2). This suggests that spiking of the compost with the polymers did not fundamentally alter the compost microbiome, even at the comparatively high loading of the compost with the test substrates. The low relative abundance of ecologically relevant taxa is often part of the difficulty when studying processes within highly active and complex environmental microbial communities (Wellington et al., 2003; Uhlik et al., 2009). Particularly soil environments, and more so thermophilic composting conditions, offer a plethora of alternative nutrients and niches, resulting in a highly diverse community, both phylogenetically and functionally. It can be expected that provision of a novel growth substrate into compost will create an additional niche to be occupied by specialized community members capable of utilizing the substrate for their growth (Goldfarb et al., 2011). However, the recalcitrant nature of LCAP, paired with availability of alternative carbon sources, is unlikely to provide a fitness advantage large enough to fundamentally alter core community dynamics. The provision of such slow-degrading substrates is more likely to result in a low steady-state abundance of community members capable of utilizing that carbon source, particularly those that are able to catalyze the initial, rate-limiting step of the mineralization process.

It has to be considered, however, that this study was based on only a single timepoint and was therefore not able follow community dynamics, which would further inform on the substrate-induced shifts, especially with regard to potential degraders and in reference to the degradation dynamics. At the time of sampling, all incubations had reached a plateau in their CO_2_ production, suggesting that active degradation of the substrates had ended and that degrader populations might have been in decline at that point, potentially resulting in low abundances or even loss of degraders (Eck et al., 2023).

According to the associated ASVs, the relative abundance of *Thermomonospora* increased significantly across all LCAP-treated composts, while *Pseudonocardia* also increased in cellulose-treated reactors. Both *Thermomonospora* and *Pseudonocardia* have previously been implicated in the degradation of PLA, and *Thermomonospora* species such as *T. curvata* are known for their ability to degrade cellulose and lignin (Stutzenberger, 1972; McCarthy, 1987; Butbunchu and Pathom-Aree, 2019). Similar functional capabilities have recently been attributed to a *Pseudonocardia* sp. strain (Sharapova, 2023). These functional associations are in line with a potential involvement in the degradation of cellulose and LCAP in the studied compost samples.

The cellulose-treated compost exhibited further taxa with significantly increased relative abundance, including ASVs associated with order R7C24 and family A4b of the phylum Verrucomicrobiota, and genus *Longispora* of the phylum Actinomycetota. While not much is known about order-R7C24 and family-A4b bacteria, *Verrucomicrobiota* include thermoacidophilic members, as well as cellulose degrading strains (Schlesner et al., 2006; Dunfield et al., 2007; Pol et al., 2007; Islam et al., 2008; Bergmann et al., 2011). While cellulose degradation has not yet been demonstrated for members of the genus *Longispora*, apart from a sequencing-based association of this genus with cellulose degradation in a composting setting, members of the same family (Micromonospora) are known to degrade cellulose (Matsumoto et al., 2003; de Menezes et al., 2008; Shiratori-Takano et al., 2011; Piao et al., 2017; Duan et al., 2022).

These results suggest that, apart from *Pseudonocardia*, various other potential cellulose-degrading taxa were present in the cellulose treatment. Interestingly, despite their reported capacity to degrade cellulose, *Thermomonospora* did not establish or maintain a competitive population in the cellulose treatments. While it is possible that *Thermomonospora* did not contribute to cellulose degradation under the conditions used, an initial population might also have declined below the detection limit following the depletion of cellulose, considering that samples were taken at the end of the degradation experiment. In contrast, *Thermomonospora*-related phylotypes were consistently enriched in the LCAP treatments, where their relative abundance exceeded that of *Pseudonocardia* by approximately fourfold. This suggests a stronger association of *Thermomonospora* with LCAP degradation, while *Pseudonocardia* may have utilized both cellulose and LCAP substrates, either directly or via secondary degradation products.

The close phylogentic relationship of the detected *Thermomonospora* ASVs to *T. curvata* DSM43183, reported to encode the thermostable polyester hydrolase Tcur1278 (Wei et al., 2014), prompted further analysis for potential functional evidence of their involvement in the observed LCAP degradation. For the identified *Pseudonocardia* ASVs, however, no close association with plastic-degrading functions could be identified.

Qualitative PCR with specific primers confirmed the presence of *tcur1278* in the LCAP-treated composts, with the exception of the PE-18,18 treatment. The lack of amplification might be of a technical nature, or suggests a different genotype in these samples, despite the same ASVs being detected as in the other LCAP-treatments. A further functional link was established by demonstrating Tcur1278 activity on PE-2,18 as a representive substrate for LCAP. Results confirmed that Tcur1278 is able to depolymerize PE-2,18, albeit at a lower rate than that observed for the monomer production from PCL as positive control. The repeat units of LCAP are significantly longer than those of PCL, resulting in fewer hydrolysable ester bonds along the polymer chains and, consequently fewer potential cleavage sites for a hydrolase enzyme. This reduced ester-bond frequency might also affect the binding and orientation of the substrate in the active site, and thereby the degradation efficiency. Additionally, the molecular weight of PCL is relatively low with ~14,000 g mol^−1^ compared to the ~80,000 g mol^−1^ of PE-2,18, likely increasing its enzymatic depolymerization by offering more end-groups and a less dense polymer-chain arrangement, increasing enzymatic accessibility (Im et al., 2023).

PE-2,18 was chosen to provide proof of concept for LCAP depolymerization due to its higher ester bond frequency compared to the other two LCAP materials, but also due to the facile detection of the depolymerization product ethylene glycol, which is readily soluble in water. In contrast, the long-chain dialcohol and diacid monomers that are released from PE-12,12, PE-18,18 and also PE-2,18, are poorly water-soluble and require solvent extraction and LC–MS-based detection (Schwab et al., 2024). While the activity of Tcur1278 was not tested on PE-12,12 and PE-18,18, their structural similarity to PE-2,18 suggests that depolymerization is likely, potentially at significantly lower efficiency due to the reduced frequency of ester bonds and possibility of increased steric hindrance from longer repeat units. This was previously observed for the commercially available cutinase enzyme of the fungus *H. insolens*, for which a significantly reduced depolymerization rate was observed with PE-18,18 as substrate compared to PE-2,18 (Eck et al., 2023). It has also been suggested that particularly the length of the diol monomer controls depolymerization rates, at least with regard to the *H. insolens* cutinase, where increased diol-length resulted in reduced depolymerization rates for LCAP (Schwab et al., 2024).

For all LCAP variants, the respective diacids and diols are non-toxic and expected to be further metabolized through established bacterial pathways. The diacids can be activated and degraded via fatty-acid *β*-oxidation, while ethylene glycol in funneled into the TCA cycle. The long-chain diols are likely oxidized to the corresponding diacids before entering fatty-acid β-oxidation. Although these pathways are well established in bacteria, future work using isolates or transcriptomics could provide direct evidence for their involvement in LCAP degradation.

Despite the functional link of Tcur1278-encoding *Thermomonospora* and LCAP degradation, our cultivation attempts using the reference strain *T. curvata* DSM43183 with LCAP powder as the sole or additional carbon and energy were unsuccessful. However, also cultivation with cellulose powder, a verified substrate, was unsuccessful under the conditions we used. Most likely we were not able to generate the cultivation conditions for inducing the expression of the required depolymerase enzymes. Various factors may influence the successful cultivation including, but are not limited to, the medium composition and the stability of individual components, the availability of specific growth factors, the dependence on other microorganisms for growth on complex polymers, or repression of depolymerase gene expression due to carbon catabolite repression or the absence of inducers. For future studies, we recommend using microcosms containing ‘live’ (non-autoclaved) compost rather than soil, to better simulate the original degradation environment and retain a functionally active microbial community. For axenic cultivation attempts, we also suggest systematic testing and optimization of individual medium components to ensure that all essential nutrients are available and that depolymerase expression is not inadvertently suppressed.

While isolation attempts were not part of this study, various thermophilic filamentous actinomycetes were previously isolated from compost as polyester plastic degraders such as *Streptomyces*, *Thermobifida* (formerly *Thermomonospora*), *Saccharomonospora*, and *Thermoactinomyces*, further supporting a high relevance of this taxon for facilitating plastic degradation in these systems (Kleeberg et al., 1998; Hu et al., 2010). Additionally, bioinformatic mining of (meta)genomes has led to the identification of various biochemically confirmed polyester degrading enzymes phylogenetically linked to actinomycetes, including Tcur1278 (Wei et al., 2014; Buchholz et al., 2022). Other than these preceding studies, however, we were able to link Tcur1278-encoding phylotypes of *Thermomonospora* to the degradation of polyester-based plastics *in situ*, rather than through enrichment-based isolation or homology-based enzyme mining, providing a rare insight into the ecology of plastic degradation in the context of thermophilic composting.

Thermophilic compost environments play a key role in waste management within a circular economy, as such, an improved understanding of the organisms influencing plastic degradation within these systems is highly relevant to improve this process. Accelerating the biodegradation of biodegradable plastics in compost is of particular interest, given these compounds are expected to be diverted from general waste streams to composting facilities. A better understanding of the degrader communities and functions within these systems can lead to development of optimized plastic-degrading microbial communities to accommodate increases in biodegradable plastic waste. As such, the identification of *T. curvata*-like phylotypes as active participants in LCAP degradation under thermophilic composting conditions has both ecological and practical implications. As these organisms appear to occupy a distinct niche associated with recalcitrant polymer breakdown, they may contribute to the poorly characterized, but frequently observed, plastic-degradation capacity of thermophilic composting environments. Additionally, the broad substrate spectrum of Tcur1278 including PET, PCL, and now LCAP, suggests a high potential for the depolymerization of various ester-linked plastics. Therefore, targeted enrichment or introduction of such organisms through bioaugmentation strategies could enhance degradation performance.

Overall, our study provides new insight into the thermophilic composting process often used as a microbial ‘black-box’ system for regulatory biodegradation studies, by combining community profiling, differential abundance analysis, and functional gene evidence with enzyme-level validation. Our study strongly suggests *Thermomonospora* species closely related to *T. curvata* as a key phylotype for the mineralization of LCAP under thermophilic composting conditions. Not only was this phylotype detectable with increased relative abundance exclusively in LCAP-mineralizing compost reactors, but the presence of *tcur1278* in these samples was confirmed, along with the enzyme’s capacity to depolymerize LCAP *in vitro*. While previous work demonstrated enzymatic activity of Tcur1278 on PET and PCL, its environmental relevance and activity on LCAP had not been investigated. Therefore, our findings not only expand the known substrate range of this enzyme but also demonstrate its presence and likely functional role in an industrial composting context. However, it is important to note that despite our comprehensive analysis, definitive evidence of the attributed role of *Thermomonospora* and Tcur1278 would require further analysis. Additionally, while we focused on *Thermomonospora* in this study, *Pseudonocardia* phylotypes should also be further examined for involvement in LCAP degradation. Future work employing correlative network-based analyses could provide deeper insight into the potential role of microbial interactions in LCAP degradation (Kumar et al., 2024). In addition, isolation attempts or cultivation independent multi-omics approaches that directly link identity and function, could help to unequivocally identify the primary LCAP degraders (Wright et al., 2021; Malik et al., 2023). Dedicated labeling approaches such as nucleic-acid stable isotope probing (DNA/RNA-SIP) in combination with sequencing analyses may provide further confidence in the ecological relevance of candidate taxa by providing direct proof of substrate-carbon assimilation, as previously done with less complex and/or difficult to obtain isotopically-labeled substrates (Luo et al., 2009; Xie et al., 2011; Uhlik et al., 2012; Vogt et al., 2016; Lerner et al., 2020), and recently, for the first time, with a plastic substrate (Odobel et al., 2025).

## Data Availability

Raw sequencing reads of the 16S rRNA gene and ITS amplicons were deposited under ENA accession number PRJEB87387.
