# Supplementary material for: Thermomonospora spp. are implicated in the biodegradation of long-chain aliphatic polyester bioplastics during thermophilic composting
Source: Front Microbiol. 2025 Oct 14;16:1671731. doi: 10.3389/fmicb.2025.1671731 (PMC12560057; doi:10.3389/fmicb.2025.1671731)
Supplement: Supplementary file 1 [file Data_Sheet_1.pdf]

Supplementary Information

Thermomonospora spp. are implicated in the biodegradation of long-chain aliphatic polyester (LCAP) bioplastics during thermophilic composting

Harry Lerner[1]*, Marcel Eck[2], Christoph Link[3], Timo Witt[3], Glauco Battagliarin[3], Stefan Mecking[2], and David Schleheck[1,4]*

[1]Microbial Ecology and Limnic Microbiology, Limnological Institute, Department of Biology, University of Konstanz, Konstanz, Germany

[2]Chemical Materials Science, Department of Chemistry, University of Konstanz, Konstanz, Germany

[3]BASF SE, PMD/GB—B001 Ludwigshafen am Rhein, Germany

[4]The Konstanz Research School Chemical Biology, University of Konstanz, Konstanz, Germany

*Shared corresponding authorship:
Harry.Lerner@uni-konstanz.de and David.Schleheck@uni-konstanz.de

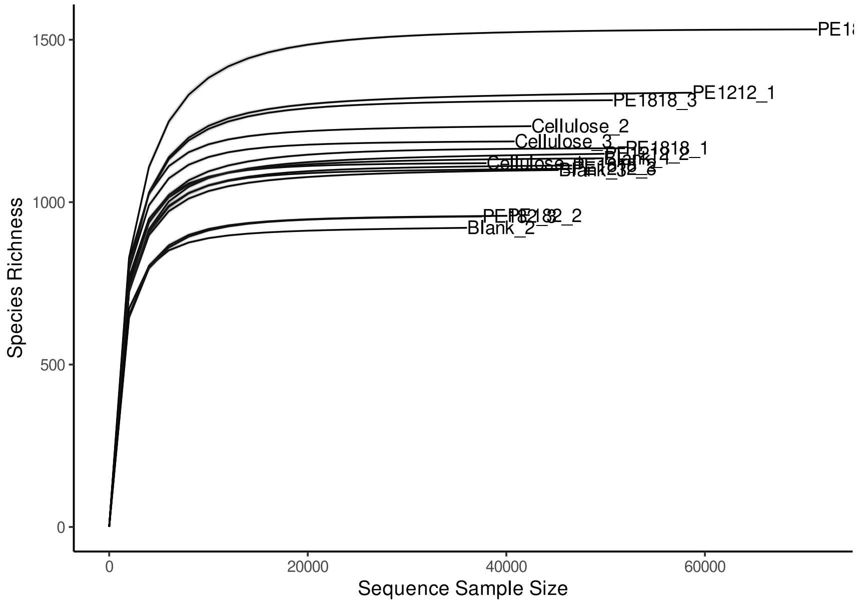

Figure S1. Rarefaction analysis of sequenced 16S rDNA amplicon libraries. Each line represents an individual replicate of the LCAP, Cellulose or Blank treatments and shows the increase in species richness with increasing number of sequenceing reads. The full asymptotes indicate a robust taxonomic sampling.

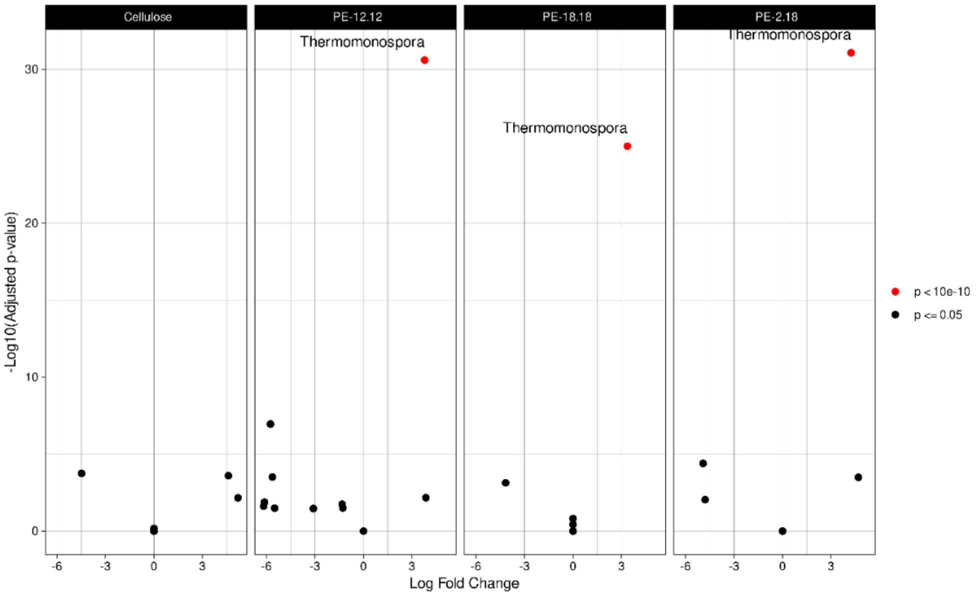

Figure S2. Visualization of negative Log10-transformed adjusted p-values for taxa in the cellulose and LCAP treatments with significant Log-fold change in abundance compared to the blanks. Taxa with significant p-values are shown as individual dots. The *Thermonospora* taxon highly significant for all three LCAP treatments is highlighted in red.

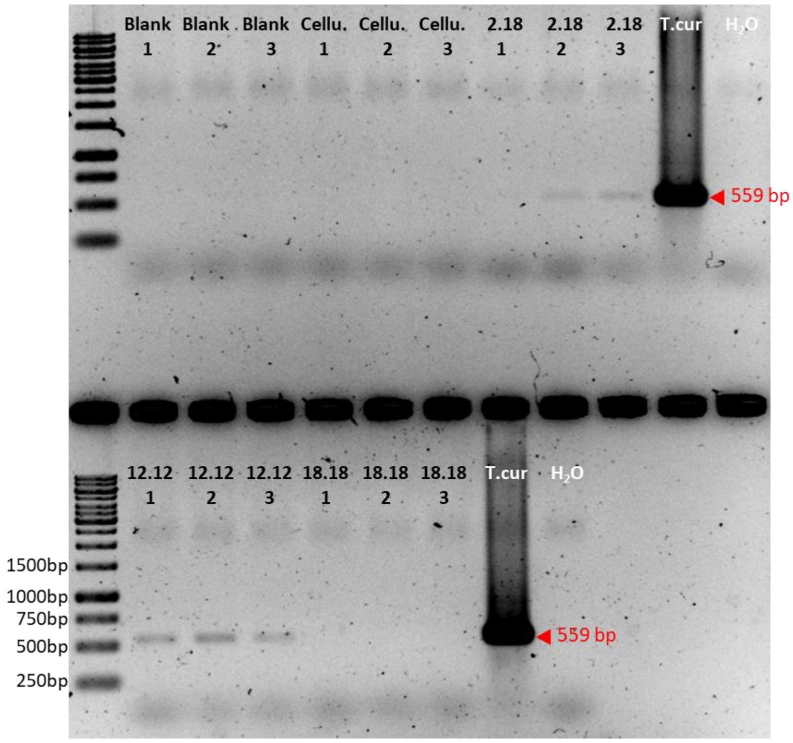

Figure S3. Agarose gel showing the amplification of the Tcur1278 gene in compost reactor samples after 30 PCR cycles. Individual lanes are labeled according to the loaded sample. A DNA marker was loaded in the first lane of each row. PCR reactions contained 5 ng of template DNA. Purified DNA from *T. curvata* DSM43183 was used as template for the positive control, while a PCR reaction with nuclease-free water in place of the DNA template served as a negative control. Bands corresponding to the Tcur1278 amplicon size are indicated by red arrows.

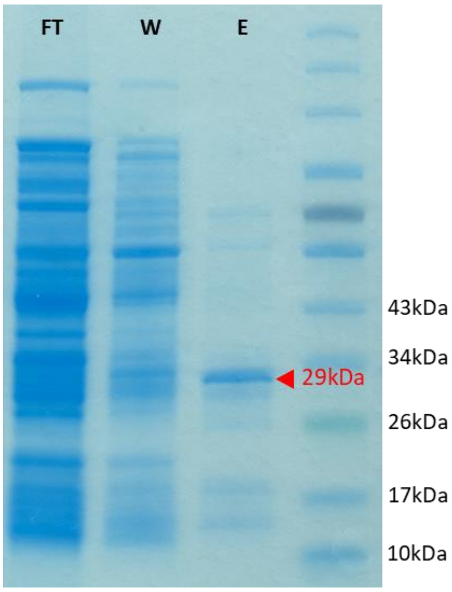

Figure S4. SDS gel showing the heterologous expression of the Tcur1278 protein in *E.coli* Rosetta Gami(DE3). The gel was loaded with protein samples obtained after the loading of the His-Trap column with cell-free lysate (flow-through, FT), following the washing of the column with 30 mM imidazole (wash, W) and the eluate obtained after applying 500 mM imidazole to the column. A protein marker was loaded in the final lane for size reference. The band corresponding to the Tcur1278 protein is indicated by a red arrow.
